# Chronic Lithium-Induced Cardiotoxicity: A Case Report and Lessons for Clinical Practice

**DOI:** 10.1155/carm/5599471

**Published:** 2025-06-26

**Authors:** Amir Heidari, Nafise Mohamadizade, Arman Hasanzade, Morteza Fazlekhoda

**Affiliations:** ^1^Department of Cardiology, Imam Hossein Hospital, Shahid Beheshti University of Medical Sciences, Tehran, Iran; ^2^Tracheal Diseases Research Center, National Research Institute of Tuberculosis and Lung Diseases, Shahid Beheshti University of Medical Sciences, Tehran, Iran; ^3^Lung Transplantation Research Center, National Research Institute of Tuberculosis and Lung Diseases, Shahid Beheshti University of Medical Sciences, Tehran, Iran

**Keywords:** cardiac block, cardiotoxicity, case report, lithium intoxication, lithium toxicity

## Abstract

**Background:** Lithium, commonly used in the treatment of bipolar disorders, is primarily known for causing neurological and renal side effects. However, cardiac side effects are rarely reported.

**Case Summary:** We present a case of chronic lithium toxicity in an 80-year-old woman. The patient was admitted to the emergency room due to loss of consciousness. Initial evaluation revealed severe sinus bradycardia as a presentation of sinus node dysfunction on the electrocardiogram, prompting the insertion of a pacemaker. During her admission to the critical care unit, it was discovered that the patient had been undergoing long-term lithium treatment without medical supervision. The serum lithium level confirmed lithium intoxication. Following the discontinuation of lithium, both neurological and cardiac manifestations of lithium toxicity resolved. After the pacemaker was removed, the patient was discharged in stable condition.

**Discussion:** Lithium has a narrow therapeutic range, which can lead to toxicity in the absence of routine monitoring. Lithium toxicity can cause serious cardiac effects and rhythm disturbances that are often overlooked because these manifestations are rare. Cardiac manifestations include arrhythmias, bradycardia, collapse, hypotension, myocardial infarction, and even death. Additionally, lithium toxicity can present with various electrocardiographic abnormalities such as T-wave inversion, sinoatrial block, PR interval prolongation, QT prolongation/dispersion, and ventricular tachyarrhythmias. Clinicians should be aware of the potential cardiac effects of lithium toxicity and consider it in patients undergoing lithium treatment. A thorough understanding of these manifestations is essential, as the wide range of symptoms can be misleading without adequate knowledge.

## 1. Introduction

Lithium is one of the most administered drugs in psychiatric patients and holds significant importance as one of the most effective medications for long-term therapy of bipolar disorders, acting as a mood stabilizer. Lithium has also been proven to have a superior preventive effect against suicide and disease relapse compared to other mood stabilizers [1–3]. However, various adverse effects have been commonly reported with this medication including diarrhea, nausea, vomiting, tremor, weakness, lightheadedness, polyuria, polydipsia, and weight gain [2, 4]. In addition to these adverse effects, lithium usage is limited due to its narrow therapeutic range (0.6–1.2 mEq/L), making close monitoring of serum levels essential. Serum levels above the therapeutic range not only result in multisystem effects, but they can also cause mortality [4].

Lithium toxicity can be categorized into acute, chronic, and acute on chronic. Studies have demonstrated that the severity of intoxication is directly related to lithium serum levels, classified as mild (1.5–2.0 mEq/L), moderate (2.0–2.5 mEq/L), and severe (over 2.5 mEq/L) [4]. While lithium toxicity, whether chronic or acute, has numerous manifestations and affects kidney, thyroid, and parathyroid function, neurological symptoms are most common [3, 4]. Neurological manifestations can vary from mild, such as weakness and tremor, to apathy, hyperreflexia, ataxia, slurred speech, and even more severe complications such as seizures, encephalopathy, and coma. Lithium toxicity can cause renal problems such as renal failure, interstitial nephritis, nephrogenic diabetes insipidus, nephrotic syndrome, or endocrine involvement, such as thyrotoxicosis, hypercalcemia, hyperparathyroidism, hypothyroidism, and euthyroid goiter [4, 5]. Although complications caused by lithium toxicity are not restricted to these problems, neurological effects alongside renal effects are the most known complications. Therefore, most physicians are not familiar with other complications of lithium toxicity, such as cardiac toxicity.

In this paper, we report a case of chronic lithium toxicity in an 80-year-old woman presenting with loss of consciousness. Initial evaluation led to the discovery of cardiac involvement, including QT interval prolongation and bradycardia. We would like to mention that this case report has been prepared in line with the CARE 2017 guideline [6].

## 2. Case Presentation

An 80-year-old female patient was admitted to our emergency room with an inability to communicate, low consciousness, and slurred speech. Her family mentioned a 2 week history of fatigue and weakness worsening over the past 2 days, along with delirium and incoherent speech. The patient had a medical history of diabetes, hyperlipidemia, and hypertension. There was no history of substance abuse, and surgical history was negative. Initial examination revealed a blood pressure of 125/70 mmHg and a pulse rate of 40 beats per minute, indicating bradycardia. Respiratory rate and temperature were normal and a Glasgow Score (GCS) of 12 indicated a loss of consciousness. Physical examination, including corneal reflex, showed no pathologic signs.

**Figure 1:**
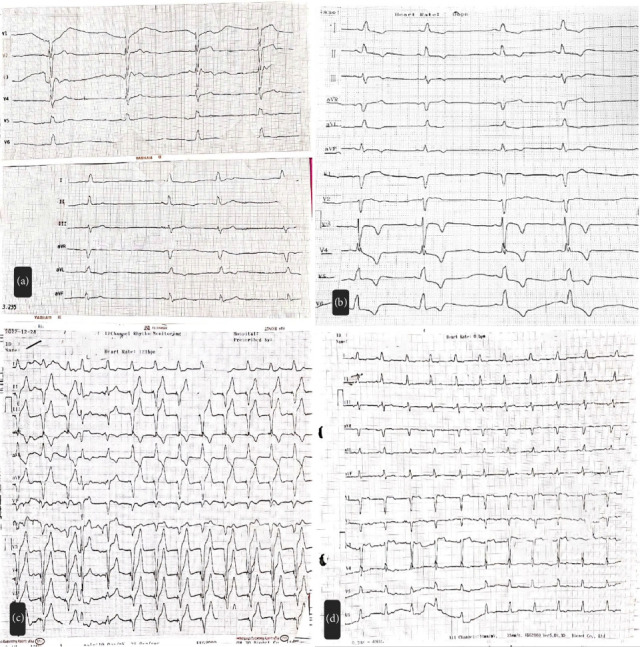
(a) (Initial presentation): ECG on emergency department admission showing severe sinus bradycardia as a result of sinus node dysfunction, long QT interval and possible bundle branch block. (b) (Catheterization lab): ECG on the same day prior to pacemaker implantation, demonstrating persistent severe sinus bradycardia with sinus node dysfunction. (c) (Post-temporary pacemaker): ECG 48 h after temporary pacemaker implantation, demonstrating VVI pacing. (d) (Recovery): ECG after pacemaker removal demonstrates complete recovery of sinus node function (regular P waves at 74 bpm, PR 160 ms).

The Electrocardiography (ECG) obtained from the patient showed sinus bradycardia as a manifestation of sinus node dysfunction (Figures 1(a), 1(b)), and based on the treating cardiologist's opinion, patient was transferred to the cardiac catheterization laboratory. A temporary external pacemaker was employed (Figure 1(c)), and the patient was transferred to the Critical Care Unit (CCU) for close monitoring. Echocardiography performed in the CCU demonstrated an ejection fraction of 50% with abnormal septal motion, though no significant valvular abnormality was detected.

During CCU hospitalization, it was discovered that the patient had been diagnosed with bipolar disorder 25 years ago following the loss of her husband. She was taking lithium at a dosage of 300 mg every 12 h since then, without supervision from a psychiatrist or any physician. High suspicion of lithium toxicity arose, confirmed by a serum level of 2.55 mEq/L, well above the therapeutic maximum serum level of 1.2 mEq/L. Moreover, laboratory results also indicated an increase in creatinine levels to 2.3. Therefore, after psychiatric and toxicology consult, the lithium was discontinued. Lithium serum level decreased in the following days getting to 2.45 mEq/L and then on the fifth day to 0.7 mEq/L. During this time the ECG showed a normal sinus rhythm with no sign of block or bradycardia (Figure 1(d)), therefore, the pacemaker was turned off and the patient was under close supervision in the CCU for a few more days. During this time there was no problem regarding patient's cardiac rhythm, indicating that the bradycardia resulted from lithium toxicity. Subsequently, the temporary pacemaker was removed, and the patient was discharged in good general health with stable vital signs and no complications. Throughout the subsequent 2 month follow-up period, the patient remained asymptomatic without any complaints.

## 3. Discussion

Lithium, widely used in psychiatry, is generally considered effective with a narrow therapeutic range and broad volume distribution (0.8 L/kg). It freely moves in blood serum without binding to plasma proteins and easily crosses the blood-brain barrier. Lithium undergoes urinary elimination, making neurological and renal complications the most common in cases of toxicity [5]. Although lithium infrequently causes cardiac complications, these manifestations are typically delayed compared to neurological effects. The delay is attributed to the progressive equilibration of lithium concentrations between extracellular and tissue compartments [7]. It is notable that strong predictors of severe toxicity in chronic lithium poisoning include the presence of diabetes insipidus, age > 50 years, hypothyroidism, and impaired renal function [8].

Clinically, evident cardiac manifestations associated with lithium toxicity include sinus node dysfunction such assinus bradycardia, sinoatrial block, and first-degree atrioventricular block, and rarely, extended QT interval, atrial flutter, atrioventricular block, right bundle department block, left anterior hemiblock, ventricular tachycardia, and ventricular fibrillation [7, 9]. Consequently, Electrocardiogram (ECG) findings related to lithium toxicity encompass a prolonged QT interval, T wave flattening and inversion, and first-degree atrioventricular (AV) conduction delay. In rare instances, ventricular tachycardia and ventricular fibrillation resulting in death have been reported [7]. Therefore, discontinuing lithium and treating cardiac manifestations of lithium toxicity is crucial. In addition to discontinuation of lithium, hemodialysis can be helpful since the renal clearance rate for lithium is about 15 mL/min which is significantly lower compared to the rate of 100 mL/min with hemodialysis [4]. Additionally, in some patients with life-threatening cardiac dysrhythmia, a temporary pacemaker and cardiac monitoring may be required until the resolution of cardiac toxicity [5, 10], similar to our case.

Sinoatrial block associated with lithium therapy was initially reported in 1975 [11]. While toxic levels of lithium are known to impact the sinus node, there are few cases where sinus bradycardia and sinus nodal disease have been reported. Subsequent reports have indicated reversible sinus node suppression as a consequence of chronic lithium therapy, even at therapeutic levels. Although the mechanism is poorly understood, it may be linked to lithium causing disturbance in the Na^+^/Ca^2+^ exchanger and Na/K pump in the membrane of cardiac cells [5, 12].

In this case, the patient exhibited bradycardia. Myocardial infarction was ruled out and despite a previous study documenting lithium-induced bradycardia as a consequence of lithium-induced hypercalcemia and hypothyroidism [13], in our case, the serum calcium level was normal, and the patient did not have hypothyroidism. Several mechanisms related to lithium-induced ECG changes were considered. Firstly, lithium's competition with sodium, potassium, calcium, and magnesium ions could disrupt fluid and salts balance. Interaction with the sodium–calcium exchanger and Na/K pump might affect cellular membrane physiology. Lithium's ability to decrease intracellular potassium concentration, replace intracellular calcium, cause hypercalcemia, and reduce depolarization rate and electrical impulse propagation induce various ECG changes, especially those related to myocardial repolarization, such as nonspecific ST segment and T wave changes [5, 14]. Secondly, lithium could induce sinus node dysfunction by blocking voltage-gated sodium channels, decreasing sensitivity of the sinus node to sympathetic stimulation, and increasing the incidence of impulse generation failure (sinus arrest), explaining the sinus arrest in this patient [14, 15].

## 4. Conclusion

Clinicians must be cautious in treating patient undergoing lithium therapy, given the narrow therapeutic range of lithium, and should be aware of the variety of manifestations of lithium intoxication, particularly cardiac manifestation, since they are life-threatening and rare. Therefore, it is important to identify at-risk patients through history taking, monitoring for side effects during therapy, and prompt referral to a specialist for prevention and treatment of intoxication. Emphasizing the importance of recognizing the delayed complications of chronic lithium poisoning is vital, as its diverse manifestations can lead to misdiagnoses.

## Data Availability

The data that support the findings of this study are available from the corresponding author upon reasonable request.
